# Ready Access to Proquinazid Haptens via Cross-Coupling Chemistry for Antibody Generation and Immunoassay Development

**DOI:** 10.1371/journal.pone.0134042

**Published:** 2015-07-27

**Authors:** Francesc A. Esteve-Turrillas, Josep V. Mercader, Javier Parra, Consuelo Agulló, Antonio Abad-Somovilla, Antonio Abad-Fuentes

**Affiliations:** 1 Department of Biotechnology, IATA-CSIC, Agustí Escardino 7, 46980 Paterna, València, Spain; 2 Department of Organic Chemistry, Universitat de València, Doctor Moliner 50, 46100 Burjassot, València, Spain; Institute for Frontier Medical Sciences, Kyoto University, JAPAN

## Abstract

Bioconjugate preparation is a fundamental step for antibody generation and immunoassay development to small chemical compounds. For analytical targets holding in their structure an aryl halogen atom, cross-coupling reactions may be a simple and efficient way to obtain functionalized derivatives; thus offering great potential to elicit robust and selective immune responses after being coupled to immunogenic carrier proteins. However, substitution of the halogen atom by an aliphatic chain might eventually compromise the affinity and specificity of the resulting antibodies. In order to address this issue, proquinazid, a new-generation fungicide with outstanding performance, was chosen as model analyte. Two functionalized derivatives differing in spacer arm rigidity were synthesized by Sonogashira cross-coupling chemistry. These haptens were covalently coupled to bovine serum albumin and the resulting immunoconjugates were employed for rabbit vaccination. Antibodies were tested for proquinazid recognition by direct and indirect competitive immunoassay, and IC_50_ values in the low nanomolar range were found, thus demonstrating the suitability of this straightforward synthetic strategy for the generation of immunoreagents to compounds bearing an aryl halide. Following antibody characterization, competitive immunoassays were developed and employed to determine proquinazid residues in grape musts, and their analytical performance was satisfactorily validated by comparison with GC–MS. Besides having described the development of the first immunochemical method for proquinazid analysis, an efficient functionalization approach for analytes comprising aryl halides is reported.

## Introduction

Antibody-based detection techniques are currently invaluable analytical tools in numerous disciplines, including basic biochemical and biomedical research, forensic toxicology, clinical diagnostics, food safety, and environmental monitoring. The huge success of these methodologies relies on the exquisite affinity and specificity that antibodies commonly exhibit towards the molecular target. While generation of this sort of binding molecules is most often straightforward for large entities, like proteins and microorganisms, production of antibodies to small organic chemicals, so-called haptens, can frequently be a challenging task. Haptens cannot elicit an immune response by themselves, so coupling to a carrier protein is mandatory in order to trigger an efficient good-quality response. Unfortunately, most interesting targets do not possess ready-to-activate chemical groups for protein conjugation, so adequate derivatives must be prepared, often by total synthesis comprising complex multi-step synthetic approaches. Moreover, even if suitable functional groups are already present in the analyte, such as -COOH, -NH_2_, -OH, and -SH, coupling through these positions may not be advisable because potentially strong antigenic determinants—immunodominant epitopes—involved in high-affinitiy and specific binding (hydrogen bonding, ionic, or electrostatic interactions, etc.) would be concealed.

Since the discovery and development of palladium-catalyzed cross-coupling reactions revolutionized organic chemistry in the early 1970s, utilization of such chemical strategies has increased exponentially both in academia and in industry. These synthetic procedures provide convenient and highly practical methods for the construction of previously difficult or impossible to generate carbon—carbon bonds [1]. Nowadays, one of the most important and widely used palladium-catalyzed cross-coupling reactions is the Sonogashira reaction, which permits the coupling of aryl or vinyl halides to terminal acetylenes under mild conditions [2]. Despite the vast potential of these reactions for introducing new functionalities at desired positions of the molecular framework of the target compound, they have hardly been exploited in immunochemistry for the synthesis of haptens ready to be used for bioconjugate preparation. In the last few years, we have reported the application of the Sonogashira cross-coupling reaction to the successful preparation of conveniently functionalized derivatives of various modern agrochemicals [3–11]. In all these cases and as a key step in each particular synthetic strategy, previous preparation of a halogenated intermediate was required because all of the concerned analytes were lacking from the proper halogen moiety for further introduction of the corresponding spacer arm via cross-coupling chemistry.

While it early became apparent to us that cross-coupling chemistry could be a valuable tool for easily affording functionalized derivatives of compounds already bearing aromatic halogen atoms—particularly iodine, bromine, and chlorine—the question arising was whether the substitution of the halogen atom—a potentially strong antigenic determinant—by an alkynyl chain or the corresponding saturated one, could compromise the affinity and specificity of the eventually derived biomolecular binders.

The past 30 years have witnessed a period of significant expansion in the use of halogenated compounds in several fields, including agrochemical research and development. A recent survey about modern agrochemicals shows that around 79% of the new active ingredients used nowadays are halogen substituted, and 12 of those products are among the 20 best-sold compounds, accounting for sales of US$ 6430 million [12]

Proquinazid is a novel quinazolinone fungicide developed by DuPont, and one of the newest incorporations to the available arsenal of active ingredients enabling to efficiently combat fungal pathogens that damage valuable crops (http://www2.dupond.com). Proquinazid was particularly effective for powdery mildew control in cereals, cucurbits, and grapevines, and it was first registered in several European countries in 2004, even though full approval and inclusion in Annex I of the European Union occurred in 2009 [13], and more recently (2012) in Australia [14]. Proquinazid is thought to act by interfering signal transduction pathways, even though the exact molecular target is unknown [15]. A remarkable chemical feature of proquinazid is the presence of an aryl iodine atom in its structure, which contributes to its excellent field performance and increased stability to sunlight, allowing field application at low rates [16]. This fact, together with its novelty and efficacy as fungicide, makes proquinazid an outstanding candidate to study the adequacy of cross-coupling reactions to the synthesis of adequate functionalized mimics that enable to trigger specific and strong immune responses and eventually afford high-affinity and specific antibodies. Additionally, to the best of our knowledge, no previous studies dealing with the generation of immunoreagents to proquinazid have been reported. Accordingly, we herein describe the synthesis of two proquinazid haptens via Sonogashira cross-coupling reaction and the evaluation of their suitability for antibody generation. In these haptens, the iodine atom of proquinazid was replaced by an alkynyl carboxylate six-carbon linker in order to examine both the effect of a rigid triple C-C bond directly linked to the derivatization site, and that of the corresponding saturated alkyl chain, which resulted in a more flexible spacer arm. The described haptens were coupled to the appropriate proteins, and the so-obtained bioconjugates were used to immunize rabbits. Finally, performance of the resulting antisera in terms of affinity, specificity, and bioanalytical capability was evaluated by competitive enzyme-linked immunosorbent assay (cELISA).

## Materials and Methods

### Reagents and instrumentation

Proquinazid (6-iodo-2-propoxy-3-propylquinazolin-4(3*H*)-one, CAS Registry number 189278-12-4, Mw 372.2) and other fungicide standards were purchased from Fluka/Riedel-de-Haën (Seelze, Germany) and Dr. Ehrenstorfer (Augsburg, Germany). Working solutions were prepared as concentrated solutions in *N*,*N*-dimethylformamide (DMF) and kept at –20°C in amber glass vials. Horseradish peroxidase (HRP), ovalbumin (OVA), *o*-phenylenediamine (OPD) and triphenyl phosphate (TPP) were purchased from Sigma/Aldrich (Madrid, Spain). Sephadex G-25 HiTrap Desalting columns from GE Healthcare (Uppsala, Sweden) were used for conjugate purification. Polyclonal goat anti-rabbit immunoglobulin peroxidase conjugate (GAR‒HRP) was from Biorad (Hercules, CA, USA). Bovine serum albumin (BSA) fraction V was purchased from Roche Applied Science (Mannheim, Germany). Adult bovine serum and Freund’s adjuvants were from Sigma/Aldrich (Madrid, Spain). Ninety-six well Costar flat-bottom high-binding polystyrene ELISA plates were from Corning (Corning, NY, USA). Ultraviolet‒visible spectra and assay absorbances were read with a PowerWave HT from BioTek Instruments (Winooski, VT, USA). ELISA plates were washed with an ELx405 microplate washer also from BioTek Instruments.

Composition, concentration, and pH of the employed buffers were: i) PB, 100 mM sodium phosphate buffer (pH 7.4); ii) PBS, 10 mM sodium phosphate buffer (pH 7.4) with 140 mM NaCl; iii) PBS-T, PBS containing 0.05% (v/v) Tween 20; iv) CB, 50mM carbonate‒bicarbonate buffer (pH 9.6); v) Washing solution, 150 mM NaCl and 0.05% (v/v) Tween 20; vi) Enzyme substrate buffer, 25 mM citrate and 62 mM sodium phosphate buffer (pH 5.4); and vii) 2xPBS-T, 20 mM sodium phosphate buffer (pH 7.4) with 280 mM NaCl and 0.05% (v/v) Tween 20.

### Hapten synthesis

#### 1 General Procedures

Anhydrous solvents were freshly distilled under nitrogen from Na or Na/benzophenone (THF, pentane) or dried over a bed of activated molecular sieves (DMF) or KOH (Et_3_N). Other solvents and reagents were obtained from commercial sources and used without purification. Reactions were monitored by thin-layer chromatography on 0.25 mm pre-coated silica gel plates. Visualization was carried out with UV light and ethanolic phosphomolybdic acid or aqueous ceric ammonium molybdate solutions. Products were purified by flash column chromatography on silica gel 60 (particle size 0.043–0.063 mm). Melting points were determined on a Büchi M-560 apparatus and are uncorrected. ^1^H/^13^C NMR spectra were recorded at 298 K in the indicated solvent on a Bruker DRX-300 (300/75 MHz) or Bruker Advance-400 (400/100 MHz) spectrometers. The chemical shifts are expressed in ppm (δ scale) relative to the residual solvent for ^1^H (CHCl_3_ at 7.26 ppm) or to the central peak of solvent ^13^C signal (CDCl_3_ at 77.0 ppm). Carbon substitution degrees were established by DEPT pulse sequences. The abbreviation used for NMR data are as follows: s, singlet; d, doublet; dd, double doublet; t, triplet; quint, quintuplet; sext, sextuplet; br, broad; m, multiplet; Qz, quinazolinone ring. Infrared (IR) spectra were obtained using a Nicolet Avatar 320 FT-IR spectrometer. High resolution mass spectra (HRMS) were recorded by the electrospray (ES) ionization mode using a Micromass VG Autospec spectrometer.

**Fig 1:**
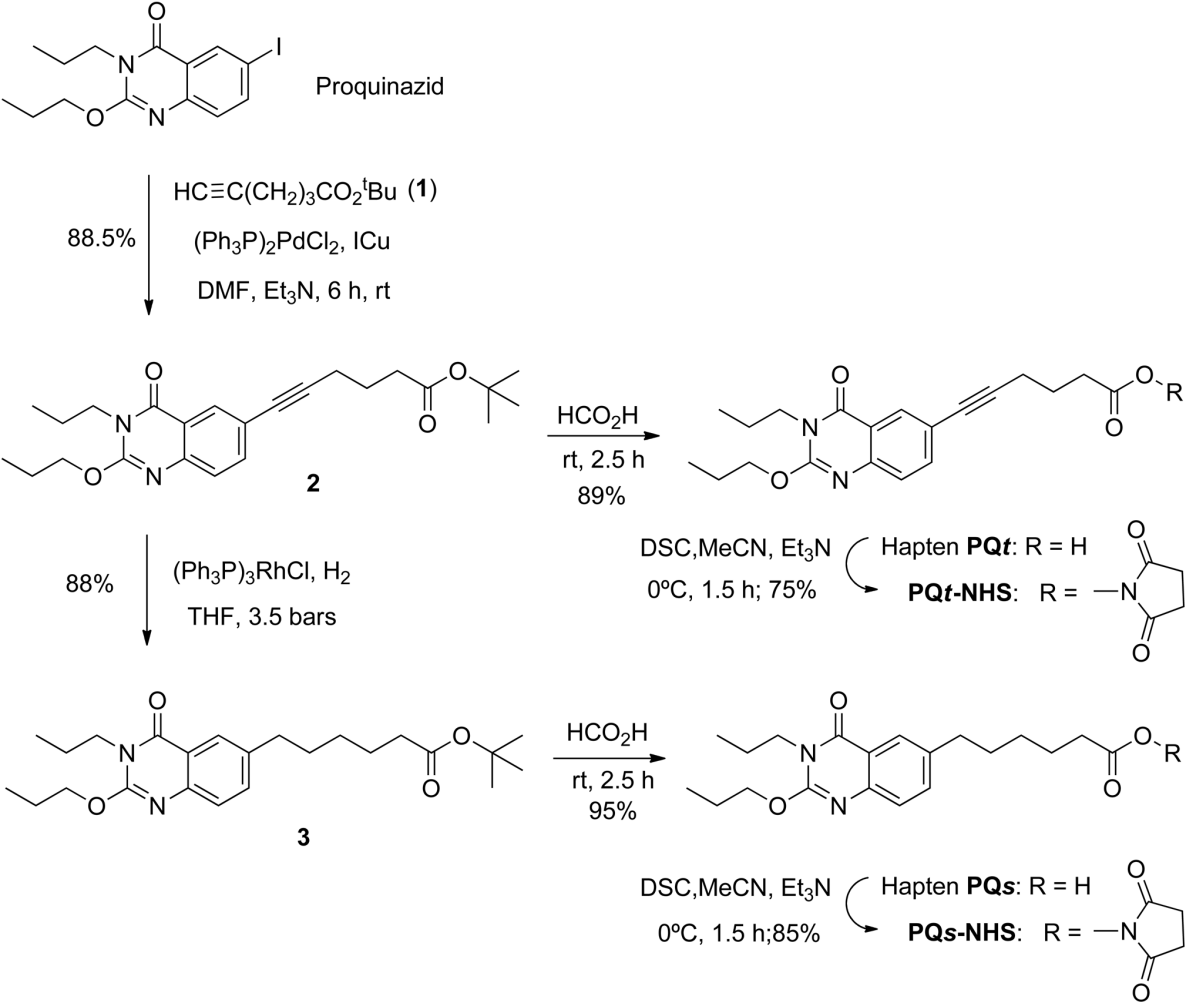
Molecular structure of proquinazid and synthetic route for the preparation of haptens PQ*s* and PQ*t* and their *N*-succinimidyl esters.

The following is a detailed description of the synthetic procedures involved in hapten preparation, as schematized in Fig 1, as well as complete spectroscopic data thereof and of the intermediates of their synthesis.

#### 2 Synthesis of hapten PQ*t*

2.1 Preparation of tert-butyl 6-(4-oxo-2-propoxy-3-propyl-3,4-dihydroquinazolin-6-yl)hex-5-ynoate (2): Dry DMF (2 mL) was added to a mixture of proquinazid (203.8 mg, 0.548 mmol), *tert*-butyl hex-5-ynoate (**1**) (138.2 mg, 0.822 mmol, 1.5 eqs), Cl_2_Pd(PPh_3_)_2_ (11.6 mg, 0.016 mmol, 0.03 eqs) and CuI (1.05 mg, 0.0054 mmol, 0.01 eqs) under nitrogen. Proquinazid was synthesized in three steps from commercially available 2-amino-5-iodobenzoic acid following a previously described procedure [17], whereas *tert*-butyl hex-5-ynoate was prepared from commercial hex-5-ynoic acid as reported by Mercader et al.[5]. The mixture was degassed through several freeze—thaw cycles, then anhydrous Et_3_N (1.6 mL) was added and the degassing-purging process was repeated twice. After stirring at room temperature for 6 h, the resulting reaction mixture was cooled down, filtered through cotton and most of the Et_3_N was removed under vacuum. The residue was diluted with ethyl acetate and washed with a 1.5% aqueous solution of LiCl and brine, dried over anhydrous Na_2_SO_4_ and concentrated under reduced pressure to produce a dark orange, viscous oil. Purification by silica gel chromatography, using hexane—CHCl_3_ 3:2 as eluent, afforded compound **2** (200 mg, 88.5%) as a brownish amorphous solid. ^1^H NMR (CDCl_3_, 300 MHz) δ 8.19 (1H, d, *J* = 1.9 Hz, H-5 Qz), 7.60 (1H, dd, *J* = 8.5 and 1.9, Hz, H-7 Qz), 7.34 (1H, d, *J* = 8.5 Hz, H-8 Qz), 4.42 (2H, t, *J* = 6.5 Hz, O*CH*_*2*_CH_2_CH_3_), 4.04 (2H, m, N*CH*_*2*_CH_2_CH_3_), 2.47 (2H, t, *J* = 7 Hz, H-4), 2.42 (2H, t, *J* = 7.6 Hz, H-2), 1.93–1.63 (6H, m, H-3, OCH_2_ *CH*_*2*_CH_3_ and NCH_2_ *CH*_*2*_CH_3_), 1.45 (9H, s, OCMe_3_), 1.05 (3H, t, *J* = 7.5 Hz, OCH_2_CH_2_ *CH*_*3*_), 0.95 (3H, t, *J* = 7.5 Hz, NCH_2_CH_2_ *CH*_*3*_); ^13^C NMR (CDCl_3_) δ 172.5 (C-1), 162.1 (C-4 Qz), 152.9 (C-2 Qz), 146.5 (C-8a Qz), 137.0 (C-7 Qz), 130.3 (C-5 Qz), 125.4 (C-8 Qz), 119.7 (C-6 Qz), 118.6 (C-4a Qz), 89.4 (C-5), 80.5 and 80.3 (C-6 and O*C*Me_3_), 70.0 (O*CH*_*2*_CH_2_CH_3_), 43.2 (N*CH*_*2*_CH_2_CH_3_), 34.4 (C-2), 28.1 (OC*Me*_*3*_), 24.0 (C-3), 22.0 (OCH_2_ *CH*_*2*_CH_3_), 21.6 (NCH_2_ *CH*_*2*_CH_3_), 18.8 (C-4), 11.3 (NCH_2_CH_2_ *CH*_*3*_), 10.5 (OCH_2_CH_2_ *CH*_*3*_); HRMS (TOF MS ES+) *m/z* calcd for C_24_H_33_N_2_O_4_ [M+H]^+^ 413.2440, found 413.2441.

2.2 Preparation of 6-(4-oxo-2-propoxy-3-propyl-3,4-dihydroquinazolin-6-yl)hex-5-ynoic acid (hapten PQ*t*): A solution of *tert*-butyl ester **2** (64 mg, 0.155 mmol) in formic acid (1 mL) was stirred at room temperature until TLC [developed with CHCl_3_] showed completion of the reaction (about 2.5 h). Benzene was added and the mixture was concentrated under reduced pressure, the residue was dissolved in benzene and concentrated again to obtain a brownish solid. Purification by chromatography, using CHCl_3_‒MeOH 96:4, afforded pure hapten PQ*t* (49.2 mg, 89%) as a white solid. Mp 113.7‒114.0°C (from benzene‒hexane). ^1^H NMR (CDCl_3_, 300 MHz) δ 10.47 (1H, br s, OH), 8.11 (1H, d, *J* = 1.9 Hz, H-5 Qz), 7.52 (1H, dd, *J* = 8.6 and 1.9 Hz, H-7 Qz), 7.27 (1H, d, *J* = 8.6 Hz, H-8 Qz), 4.34 (2H, t, *J* = 6.6 Hz, O*CH*_*2*_CH_2_CH_3_), 3.96 (2H, m, N*CH*_*2*_CH_2_CH_3_), 2.49 (2H, t, *J* = 7.4 Hz, H-4), 2.44 (2H, t, *J* = 7 Hz, H-2), 1.86 (2H, quint, *J* = 7.2 Hz, H-3), 1.75 (2H, sext, *J* = 7.4 Hz, OCH_2_ *CH*_*2*_CH_3_), 1.62 (2H, sext, *J* = 7.5 Hz, N*CH*_*2*_CH_2_CH_3_), 0.97 (3H, t, *J* = 7.4 Hz, OCH_2_CH_2_ *CH*_*3*_), 0.87 (3H, t, *J* = 7.5 Hz, OCH_2_CH_2_ *CH*_*3*_); ^13^C NMR (CDCl_3_) δ 178.8 (C-1), 162.2 (C-4 Qz), 152.9 (C-2 Qz), 146.5 (C-9 Qz), 137.0 (C-7 Qz), 130.3 (C-5 Qz), 125.4 (C-8 Qz), 119.6 (C-6 Qz), 118.6 (C-10 Qz), 89.0 (C-5), 80.9 (C-6), 70.1 (O*CH*_*2*_CH_2_CH_3_), 43.2 (N*CH*_*2*_CH_2_CH_3_), 32.8 (C-2), 23.6 (C-3), 22.0 (OCH_2_ *CH*_*2*_CH_3_), 21.6 (NCH_2_ *CH*_*2*_CH_3_), 18.8 (C-3), 11.3 (NCH_2_CH_2_ *CH*_*3*_), 10.5 (OCH_2_CH_2_ *CH*_*3*_); IR (KBr) *ν*_max_/cm^−1^ 3400–2400, 2958, 2925, 2854, 2230, 1707, 1701, 1596; HRMS (TOF MS ES+) *m/z* calcd for C_20_H_25_N_2_O_4_ [M+H]^+^ 357.1814, found 357.1817.

#### 3 Synthesis of hapten PQ*s*

3.1 Preparation of tert-butyl 6-(4-oxo-2-propoxy-3-propyl-3,4-dihydroquinazolin-6-yl)hexanoate (3): A solution of alkyne **2** (81.5 mg, 0.197 mmol) and Wilkinson’s catalyst (5.5 mg, 0.006 mmol, 3%) in anhydrous THF (2 mL) was evacuated and purged under an atmosphere of hydrogen gas. The hydrogen pressure was regulated to 3.5 atmospheres and the reaction mixture was stirred at room temperature overnight. The mixture was concentrated to dryness under reduced pressure to afford a brownish viscous oil that was purified by chromatography, using hexane—EtOAc 9:1 as eluent, to furnish compound **3** (72.5 mg, 88%) as a colorless oil. ^1^H NMR (CDCl_3_) δ 7.96 (1H, dd, *J* = 2 and 0.4 Hz, H-5 Qz), 7.45 (1H, dd, *J* = 8.4 and 2 Hz, H-7 Qz), 7.36 (1H, dd *J* = 8.4 and 0.4 Hz, H-8 Qz), 4.41 (2H, t, *J* = 6.5 Hz, O*CH*_*2*_CH_2_CH_3_), 4.06 (2H, m, N*CH*_*2*_CH_2_CH_3_), 2.68 (2H, t, *J* = 7.5 Hz, H-6), 2.19 (2H, t, *J* = 7.4 Hz, H-2), 1.84 (2H, quint, *J* = 7.4 Hz, H-3), 1.75-1.55 (6H, m, H-5, OCH_2_ *CH*_*2*_CH_3_ and NCH_2_ *CH*_*2*_CH_3_), 1.42 (9H, s, OC*Me*_*3*_), 1.35 (2H, m, H-4), 1.05 (3H, t, *J* = 7.4 Hz, OCH_2_CH_2_ *CH*_*3*_), 0.96 (3H, t, *J* = 7.5 Hz, NCH_2_CH_2_ *CH*_*3*_); ^13^C NMR (CDCl_3_) δ 173.1 (C-1), 163.9 (C-4 Qz), 152.3 (C-2 Qz), 145.3 (C-8a Qz), 138.8 (C-6 Qz), 134.9 (C-7 Qz), 126.0 (C-5 Qz), 125.3 (C-8 Qz), 118.5 (C-4a Qz), 79.9 (O*C*Me_3_), 69.8 (O*CH*_*2*_CH_2_CH_3_), 43.1 (N*CH*_2_CH_2_CH_3_), 35.5 (C-6), 35.3 (C-2), 31.0 (C-3), 29.6 (C-4), 28.1 (OC*Me*_*3*_), 24.9 (C-5), 22.0 (OCH_2_ *CH*_*2*_CH_3_), 21.7 (NCH_2_ *CH*_*2*_CH_3_), 11.3 (NCH_2_CH_2_ *CH*_*3*_), 10.5 (OCH_2_CH_2_ *CH*_*3*_); IR (NaCl) *ν*_max_/cm^−1^ 2959, 2874, 1736, 1705, 1654, 1597, 1485, 1456, 1351, 1300, 1179, 811, 754; HRMS (TOF MS ES+) *m/z* calcd for C_24_H_37_N_2_O_4_ [M+H)]^+^ 417.2753, found 417.2747.

3.2 Preparation of 6-(4-oxo-2-propoxy-3-propyl-3,4-dihydroquinazolin-6-yl)hexanoic acid (hapten PQ*s*): A solution of the *tert*-butyl ester **3** (72.1 mg, 0.173 mmol) in formic acid (1.2 mL) was stirred at room temperature for 2.5 h. The reaction mixture was diluted with benzene and concentrated under vacuum to give hapten PQ*s* (59 mg, 95%) as a white solid. Mp 124.7‒125.7°C (from EtOAc). ^1^H NMR (CDCl_3_) δ 9.80 (1H, br s, OH), 7.97 (1H, d, *J* = 2 Hz, H-5 Qz), 7.45 (1H, dd, *J* = 2, 8.4 Hz, H-7 Qz), 7.37 (1H, d, *J* = 8.4 Hz, H-8 Qz), 4.42 (2H, t, *J* = 6.5 Hz, O*CH*_*2*_CH_2_CH_3_), 4.06 (2H, m, N*CH*_*2*_CH_2_CH_3_), 2.69 (2H, t, *J* = 7.5 Hz, H-6), 2.34 (2H, t, *J* = 7.7 Hz, H-2), 1.84 (2H, sext, *J* = 7.4 Hz, H-5), 1.76–1.60 (6H, m, H-3, OCH_2_ *CH*_*2*_CH_3_ and NCH_2_ *CH*_*2*_CH_3_), 1.42 (2H, m, H-4), 1.06 (3H, t, *J* = 7.4 Hz, OCH_2_CH_2_ *CH*_*3*_), 0.97 (3H, t, *J* = 7.4 Hz, OCH_2_CH_2_ *CH*_*3*_). ^13^C NMR (CDCl_3_) δ 163.0 (C-4 Qz), 152.3 (C-2 Qz), 145.4 (C-8a Qz), 138.6 (C-6 Qz), 135.0 (C-7 Qz), 126.0 (C-5 Qz), 125.3 (C-8 Qz), 118.5 (C-4a Qz), 69.8 (O*CH*_*2*_CH_2_CH_3_), 43.2 (N*CH*_2_CH_2_CH_3_), 35.1 (C-6), 30.9 (C-2), 28.5 (C-5), 28.1 (C-4), 24.5 (C-3), 22.0 (OCH_2_ *CH*_*2*_CH_3_), 21.7 (NCH_2_ *CH*_*2*_CH_3_), 11.3 (NCH_2_CH_2_ *CH*_*3*_), 10.5 (OCH_2_CH_2_ *CH*_*3*_); IR (KBr) *ν*_max_/cm^−1^ 3300–2400, 2966, 2933, 2873, 1713, 1683, 1602, 1492, 1171, 936; HRMS (TOF MS ES+) *m/z* calcd for C_20_H_29_N_2_O_4_ [M+H)]^+^ 361.2127, found 361.2130.

### Synthesis of the *N*-succinimidyl esters of haptens PQ*t* and PQ*s*

Activation of the free carboxylate group of haptens PQ*t* and PQ*s* with *N*,*N’*-disuccinimidyl carbonate (DSC) and purification of the corresponding succinimidyl active ester was done as follows: the hapten (*ca*. 21 mg, 0.058 mmol) and DSC (19.3 mg, 0.075 mmol, 1.3 eqs.) were dissolved in dry acetonitrile (1.0 mL) under nitrogen atmosphere at 0°C and treated with anhydrous Et_3_N (23 mg, 31 μL, 0.227 mmol, 3.8 eqs.). The reaction mixture was stirred at 0°C until complete consumption of starting material (as observed by TLC, about 1.5 to 2 hours). The reaction mixture was concentrated under reduced pressure without heating to give an orange oily residue that was purified by column chromatography, using CHCl_3_‒EtOAc 9:1 as eluent, to afford the *N*-succinimidyl ester of the hapten (PQ*t*-NHS or PQ*s*-NHS) in good yield (75‒85%) and purity, as evidenced from the corresponding ^1^H NMR spectrum.

^*1*^ *H NMR of activated hapten PQt-NHS* (CDCl_3_, 400 MHz): δ 8.20 (1H, d, *J* = 2 and 0.5 Hz, H-5 Qz), 7.62 (1H, dd, *J* = 8.4 and 2, Hz, H-7 Qz), 7.36 (1H, dd *J* = 8.4 and 0.5 Hz, H-8 Qz), 4.43 (2H, t, *J* = 6.6 Hz, O*CH*_*2*_CH_2_CH_3_), 4.05 (2H, m, N*CH*_*2*_CH_2_CH_3_), 2.85 (4H, br s, OCCH_2_CH_2_CO), 2.83 (2H, t, *J* = 7.4 Hz, H-4), 2.59 (2H, t, *J* = 6.9 Hz, H-2), 2.06 (2H, quint, *J* = 7 Hz, H-3), 1.84 (2H, sext, *J* = 7 Hz, OCH_2_ *CH*_*2*_CH_3_), 1.70 (2H, sext, *J* = 7 Hz, NCH_2_ *CH*_*2*_CH_3_), 1.06 (3H, t, *J* = 7.4 Hz, OCH_2_CH_2_ *CH*_*3*_), 0.96 (3H, t, *J* = 7.5 Hz, NCH_2_CH_2_ *CH*_*3*_).

^*1*^ *H NMR of activated hapten PQs-NHS* (CDCl_3_, 300 MHz): ^1^H NMR (CDCl_3_) δ 7.96 (1H, d, *J* = 2 Hz, H-5 Qz), 7.46 (1H, dd, *J* = 8.4 and 2 Hz, H-7 Qz), 7.37 (1H, d *J* = 8.4 Hz, H-8 Qz), 4.42 (2H, t, *J* = 6.6 Hz, O*CH*_*2*_CH_2_CH_3_), 4.06 (2H, m, N*CH*_*2*_CH_2_CH_3_), 2.83 (4H, br s, OCCH_2_CH_2_CO), 2.71 (2H, t, *J* = 7.4 Hz, H-6), 2.59 (2H, t, *J* = 7.4 Hz, H-2), 1.9–1.6 (8H, m, H-3, H-5, OCH_2_ *CH*_*2*_CH_3_ and NCH_2_ *CH*_*2*_CH_3_), 1.45 (2H, m, H-4), 1.06 (3H, t, *J* = 7.4 Hz, OCH_2_CH_2_ *CH*_*3*_), 0.96 (3H, t, *J* = 7.5 Hz, NCH_2_CH_2_ *CH*_*3*_).

### Preparation of hapten‒protein conjugates

Different conjugates were prepared by reaction of the purified *N*-succinimidyl ester of each hapten (PQ*t* and PQ*s*) with the free amine groups of three carrier proteins: BSA, HRP, and OVA. The final hapten-to-protein molar ratio (MR) was calculated using the absorbance values of the conjugate by assuming that the molar absorption coefficients of the hapten and the protein were the same for both the free and the conjugated forms.

1. Immunizing conjugates. BSA—hapten conjugates were prepared using 200μL of a 50 mM solution of the *N*-succinimidyl ester of the hapten (PQ*t* or PQ*s*) in anhydrous DMF. Briefly, the active ester solution was added drop wise over a 15 mg/mL BSA solution (2 mL) in CB and the mixture was stirred at room temperature in amber glass vials during 4 h. Conjugates were purified by Sephadex G-25 gel filtration using PB as eluent. The collected volume was brought to 30 mL with PB and the conjugate was stored at −20°C. The calculated final MR of both immunizing conjugates was 12.
2. Coating conjugates. OVA—hapten conjugates were prepared by adding drop wise 200μL of a 50 mM solution of the *N*-succinimidyl ester of the hapten (PQ*t* or PQ*s*) to 2 mL of a 15 mg/mL OVA solution in CB under stirring. The reaction was mixed at room temperature during 2.5 h in amber glass vials and the conjugates were purified as before and stored at −20°C. The final MR was 7 and 10 for PQ*t* and PQ*s* haptens, respectively.
3. Enzyme tracers. HRP—hapten conjugates were prepared by adding drop wise 100μL of a 5 mM solution of the *N*-succinimidyl ester of the hapten (PQ*t* or PQ*s*) to 1mL of a 2.2 mg/mL HRP solution in CB under stirring. The coupling reaction was allowed to proceed during 4 h at room temperature with stirring in amber glass vials. Then, the conjugates were purified by gel chromatography and the pooled fractions were brought to 1 mg/mL with PB, and then diluted 1/2 with PBS containing 1% (w/v) BSA and 0.01% (w/v) thimerosal. Several aliquots of the enzyme tracer conjugate were stored at −20°C in amber vials, except the working solution which was kept at 4°C. The final MR was 2.5 for both HRP conjugates.

### Production of antibodies

Two female New Zealand white rabbits were independently immunized by subcutaneous injection with 0.3 mg of immunizing conjugate (BSA—PQ*t* or BSA—PQ*s*) in 1 mL of a 1:1 mixture of sterile PB and complete Freund’s adjuvant. Animals were boosted at 21-day intervals with the same immunogen suspended in a mixture of 0.5 mL of sterile PB and 0.5 mL of incomplete Freund’s adjuvant. Ten days after the fourth injection, rabbits were anaesthetised with xylazine/ketamine and euthanized by intracardiac puncture followed by an overdose of pentobarbital. All efforts were made to minimize suffering. Blood samples were allowed to coagulate overnight at 4°C. Then, the serum was separated by centrifugation and precipitated with a solution of saturated ammonium sulphate. This procedure was repeated again and the precipitates were stored at 4°C. This study was carried out in strict accordance with the recommendations in the European Directive 2010/63/EU concerning the protection of animals used for scientific purposes. The protocol was approved by the Ethics Committee of the Universitat de València (permit number: A1329731961154).

### Antibody-coated direct cELISA

ELISA plates were coated with 100 μL of antiserum diluted in CB, and plates were incubated overnight at room temperature. Coated plates were washed four times with washing solution and they received 50 μL per well of analyte standard in PBS plus 50 μL per well of enzyme tracer solution in PBS-T. The competitive reaction was carried out at room temperature for 1 h, and then plates were washed as described above. Finally, signal was produced by addition of 100 μL per well of freshly prepared 2 g/L OPD solution containing 0.012% (v/v) H_2_O_2_ in enzyme substrate buffer. The enzymatic reaction was stopped after 10 min at room temperature by adding 100 μL per well of 1 M sulphuric acid. The absorbance was immediately read at 492 nm with a reference wavelength at 650 nm.

### Conjugate-coated indirect cELISA

Microplates were coated with 100 μL per well of coating conjugate solution in CB by overnight incubation at room temperature. Coated microwells were washed as described and then received 50 μL per well of analyte in PBS plus 50 μL per well of antiserum diluted in PBS-T. The competitive reaction was carried out at room temperature for 1 h, and plates were washed again. Next, 100 μL per well of a 1/10^4^ dilution of GAR—HRP conjugate in PBS-T containing 10% adult bovine serum was added, and plates were incubated 1 h at room temperature. After washing the plates as before, signal was generated and the plates were read as described for the direct cELISA.

### Data treatment

Eight-point standard curves, including a blank, were prepared by five-fold serial dilution in PBS from a 1 g/L proquinazid stock solution. Experimental values were fitted to a four-parameter logistic equation using the SigmaPlot software package from SPSS Inc. (Chicago, IL, USA). Assay sensitivity was defined as the concentration of analyte at the inflection point of the fitted curve, typically corresponding to a 50% inhibition (IC_50_) of the maximum absorbance (A_max_) provided that the background signal approaches to zero.

### Buffer composition studies

Two immunoassays were selected using each of the two evaluated cELISA formats. In the direct assay, plates were coated with 1/10^4^ dilution of antibody PQ*t*#2, and a 15 ng/mL solution of tracer HRP‒PQ*t* was used in the competitive step. In the indirect cELISA, plates were coated with 0.1 μg/mL OVA—PQ*s* conjugate solution and the antibody PQ*t*#2 was diluted 1/6x10^4^ for competition.

For solvent studies, analyte standard curves were prepared in water containing between 0.5 and 10% of methanol, ethanol, acetonitrile, or acetone, and immunoreagents were prepared in 2×PBS-T. The influence of the buffer ionic strength and the pH was evaluated following a central composite design, consisting of a two-level full factorial design (α = 1.414) with 2 factors and 3 replicates that included 12 cube, 12 axial, and 15 centre points, and involving a total of 39 randomized buffer studies [18]. Proquinazid standard curves were prepared in water and they were mixed as described above with tracer or antibody solutions prepared in every of the studied buffers. The A_max_ and IC_50_ values of the inhibition curves were employed as response values and fitted to a multiple regression equation, including curvature and interaction terms, using Minitab 14.1 software (Minitab Inc., State College, PA, USA).

### Reference procedure

A QuEChERS dispersive kit (Agilent Technologies, Santa Clara, CA, USA) was employed for the extraction and purification of proquinazid residues from must samples (European Committee for Standardization Standard Method EN 15662) [19]. Purified extracts were filtered through 0.22 μm Teflon filters and analyzed by gas chromatography‒mass spectrometry (GC—MS) using an Agilent 6890N GC network system, equipped with a 7683 series autosampler, a HP-5MS (30 m × 0.25 mm × 0.25 μm) capillary column, and a 5973 mass selective detector. GC—MS measurement conditions were as follow: one microliter extract was injected in splitless mode at 300°C by employing helium as carrier with a constant flow of 1 mL/min. The oven temperature program (150°C) was held for 1 min, increased at a rate of 10°C/min up to 280°C and held at this temperature for 2 min. The transfer line and source temperatures were 280°C and 250°C, respectively. Electron impact ionization at 70 eV was used, and the employed quantification ions were 330 and 288 m/z for proquinazid and 325 and 326 m/z for TPP, the internal standard.

## Results and Discussion

### Synthesis of haptens and preparation of bioconjugates

Hapten synthesis is usually deemed the key step in the generation of antibodies to small organic chemicals, so easy access to suitable functionalized derivatives of target compounds greatly facilitates the production of anti-hapten antibodies and the development of immunochemical methods. If available, functional groups already present in the target molecule, commonly consisting in—SH,–OH,–NH_2_, and—COOH, constitute a simple and attractive approach to prepare functionalized derivatives, even though coupling through these moieties may sometimes be unwise in terms of antibody affinity and/or specificity. In 2010, the Nobel Prize in Chemistry was awarded jointly to Richard F. Heck, Ei-ichi Negishi, and Akira Suzuki for their roles in discovering and developing highly practical methodologies for C—C bond construction. A particular type of these palladium-catalyzed cross couplings is the so-called Sonogashira reaction, which allows formation under mild conditions of a C—C bond between a terminal alkyne and an aryl or vinyl halide by making use of both palladium and copper catalysts. Accordingly, this sort of reaction enables the facile introduction of a spacer arm in compounds that, like proquinazid, bear an aryl halide in their structure.

Following this approach (Fig 1), an acetylenic derivative of proquinazid (**2**) was prepared directly from the agrochemical molecule by easy substitution of the iodine atom at the C-6 position of the 4(3H)-quinazolinone ring for the *tert*-butyl ester of hex-5-ynoic acid (**1**) in the presence of catalytic amounts of dichloro-bis(triphenylphosphine)palladium (II) and copper (I) iodide at room temperature. The synthesis of hapten PQ*t* was readily completed by acid hydrolysis of the *tert*-butyl ester moiety of **2** using formic acid at room temperature. The overall yield for the transformation of proquinazid into hapten PQ*t* through this two-step sequence was *ca*. 78%. Concerning hapten PQ*s*, which has a completely saturated hydrocarbon spacer arm at the same position of the quinazolinone ring, it was straightforward prepared from intermediate **2** in two steps. First, homogeneous catalytic hydrogenation of the triple bond using Wilkinson's catalyst produced compound **3**, which was followed by mild acid hydrolysis of the *tert*-butyl ester moiety to a carboxylic acid group. The global yield for proquinazid transformation into hapten PQ*s* via this three-step reaction sequence was around 74%.

Activation of the carboxyl moiety of both haptens was easily accomplished using DSC, a procedure that allows obtaining the *N*-succinimidyl ester derivative of the haptens, *i*.*e*. compounds PQ*t*-NHS and PQ*s*-NHS (Fig 1), in high yield. Following purification by conventional column chromatography, the activated haptens were coupled to BSA (immunizing conjugates) and to OVA and HRP (assay conjugates) with precise hapten-to-protein ratios and avoiding the formation of undesired secondary by-products [4].

### Evaluation of the immune response

#### Antibody affinity

The purpose of preparing two haptens—one with a spacer arm containing a triple blond (hapten PQ*t*) and the other one with a fully saturated hydrocarbon chain (hapten PQ*s*)–was to study the effect of linker flexibility on the binding properties of the so-derived antibodies. Two antisera were obtained from each immunizing hapten; those raised from the conjugate of hapten PQ*s* were named PQ*s*#1 and PQ*s*#2, and those generated from hapten PQ*t* were PQ*t*#1 and PQ*t*#2. The ability of each antibody to recognize bioconjugates (OVA—hapten and HRP—hapten) and the free analyte was evaluated by checkerboard competitive screening analysis using both direct and indirect cELISAs. For the direct format, plates were coated with 1/10^4^ and 1/3×10^4^ dilutions of the antiserum, and next day a range of enzyme tracer concentrations (from 3 to 100 μg/L) was assayed under competitive conditions, *i*.*e*., in the presence of a series of proquinazid standard solutions. For the indirect format, plates were coated with 0.1 and 1.0 mg/L OVA—hapten conjugate, and a range of antiserum dilutions (from 1/10^4^ to 1/10^6^) were employed in the competitive step. The performance of each antibody was assessed using both homologous—the hapten in the assay conjugate was the same used to generate the antibody—and heterologous conjugates [20]. The outcome of this study was a collection of inhibition curves for each pair of immunoreagent combinations, and the parameters (A_max_, slope, and IC_50_ value) from the best inhibition curve (A_max_ over 0.8 and lower IC_50_ value) for each combination are shown in Table 1 for the direct assay and in Table 2 for the indirect assay.

**Table 1:** Assay parameters from checkerboard direct cELISA.

Antibody	HRP—hapten	[Tracer](μg/L)	Antibody dilution	A_max_	Slope	IC_50_(μg/L)
PQ*s*#1	PQ*s*	10	10^4^	1.37	0.75	33
	PQ*t*	30	10^4^	1.35	0.87	38
PQ*s*#2	PQ*s*	30	10^4^	1.22	0.81	47
	PQ*t*	100	10^4^	1.08	0.77	21
PQ*t*#1	PQ*s*	100	10^4^	--^a^	--	--
	PQ*t*	100	10^4^	0.81	0.88	20
PQ*t*#2	PQ*s*	10	10^4^	1.13	0.83	7
	PQ*t*	10	10^4^	1.02	0.81	4

^a^ This tracer was insufficiently recognized by the antibody (A_max_<0.5)

**Table 2:** Assay parameters from checkerboard indirect cELISA.

Antibody	OVA—hapten	[Conjugate](μg/L)	Antibody dilution	A_max_	Slope	IC_50_(μg/L)
PQ*s*#1	PQ*s*	100	3×10^4^	0.97	0.80	75
	PQ*t*	1000	10^5^	0.78	0.63	89
PQ*s*#2	PQ*s*	100	3×10^4^	1.11	0.88	81
	PQ*t*	100	3×10^4^	0.76	0.71	43
PQ*t*#1	PQ*s*	100	10^4^	1.06	0.60	41
	PQ*t*	100	10^4^	1.17	0.66	50
PQ*t*#2	PQ*s*	100	10^5^	0.95	0.71	10
	PQ*t*	100	10^5^	1.12	0.76	13

In the direct format, coating plates with antibodies at a 1/3×10^4^ dilution resulted in satisfactory inhibition curves, whereas a higher antibody dilution brought about very low signals—with the only exception of antibody PQ*t*#1 which recognized both the homologous and the heterologous enzyme tracers, even though not significant differences in IC_50_ values were observed by changing the assay hapten. In the indirect format, antibodies recognized both coating conjugates, and inhibition curves with sufficient signal were obtained with all immunoreagent combinations.

**Fig 2:**
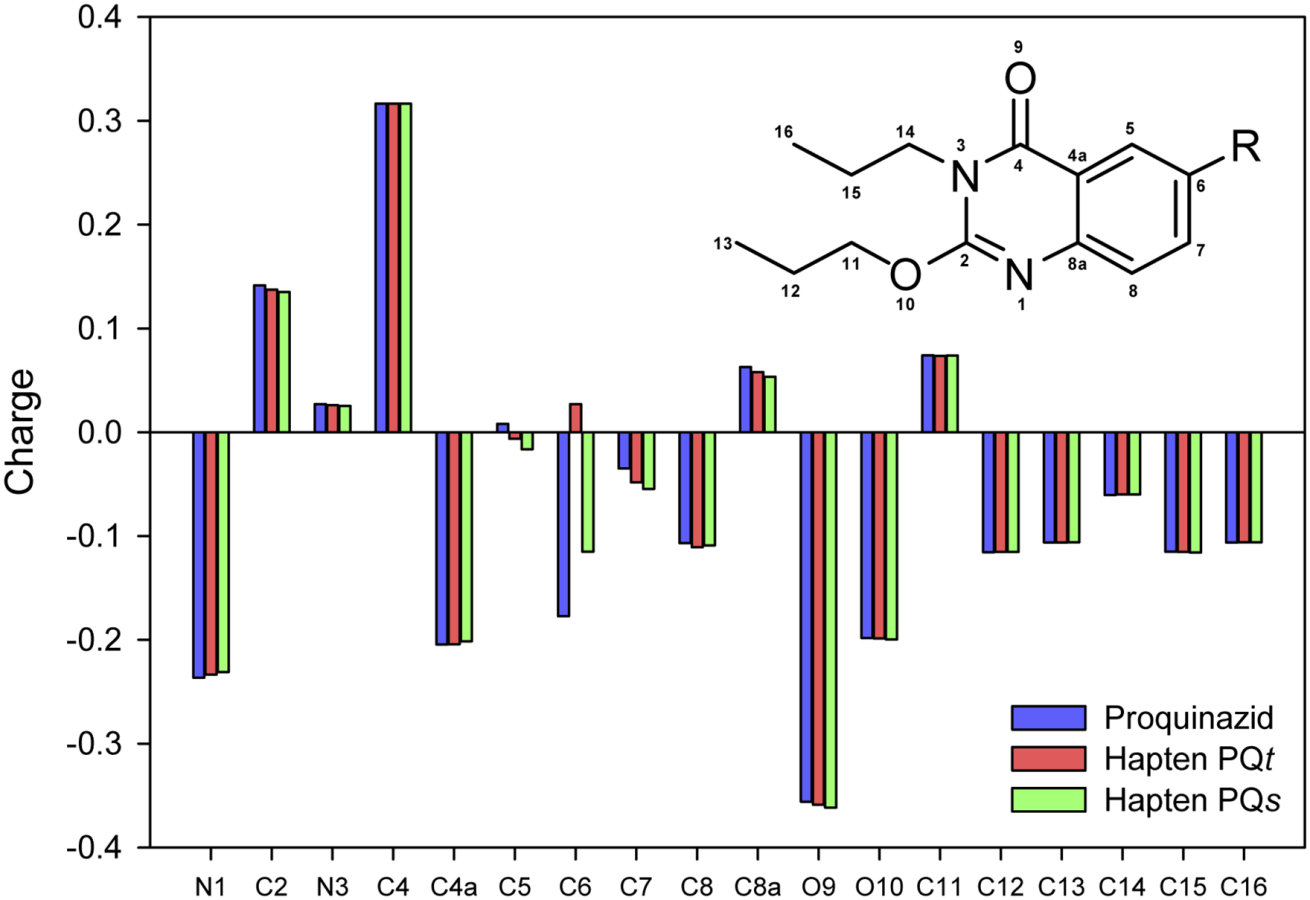
Partial charges on the atoms in proquinazid and haptens PQ*s* and PQ*t*. All semi-empirical calculations were performed using the WinMopac 2007 parametric method 3 (PM3) with the MO-G for SCIGRESS program [MO-G Version 1.1, Fujitsu Limited, Tokyo, Japan (2008)]. All initial structures were optimized first by molecular mechanics method (MM3).

These results provided compelling evidences of the usefulness of cross-coupling reactions to synthesize proquinazid haptens adequately mimicking the analyte structure, and therefore being appropriate for the production of valuable high-affinity anti-proquinazid antibodies. Regarding the nature of the hydrocarbon spacer arm, results were not clearly conclusive. Globally, the antibodies with the highest affinity to proquinazid were elicited by the hapten bearing the more rigid unsaturated linker chain (hapten PQ*t*), despite the fact that the replacement of the iodine atom by the triple bond produced a greater modification of the electronic distribution of the dihydroquinazolinone ring atoms—particularly on atom C-5 where the linker was attached—due to resonance effects (Fig 2). The most sensitive combinations were those based on antiserum PQ*t*#2, either with the homologous enzyme tracer HRP—PQ*t* in the direct format (IC_50_ = 4 μg/L) or with the heterologous coating antigen OVA—PQ*s* in the indirect assay (IC_50_ = 10 μg/L).

#### Antibody specificity

Cross-reactivity studies were performed in order to find whether other related compounds could be recognized by the anti-proquinazid antibodies. With this aim, concentrations in the 10^−2^ nM to 10^4^ nM range of 22 widely used fungicides were assayed: azoxystrobin, pyraclostrobin, dimoxystrobin, picoxystrobin, kresoxim-methyl, trifloxystrobin, fluoxastrobin, fenhexamid, tebuconazole, procimidone, cyazofamid, fenamidone, imidacloprid, tolylfluanid, fludioxonil, boscalid, pyrimethanil, mepanipyrim, cyprodinil, fenpropimorph, propiconazol, and epoxiconazol. It could be concluded that the antibodies were highly specific to proquinazid because no inhibition was observed by any of the evaluated pesticides at the studied concentrations.

### Immunoassay characterization

**Fig 3:**
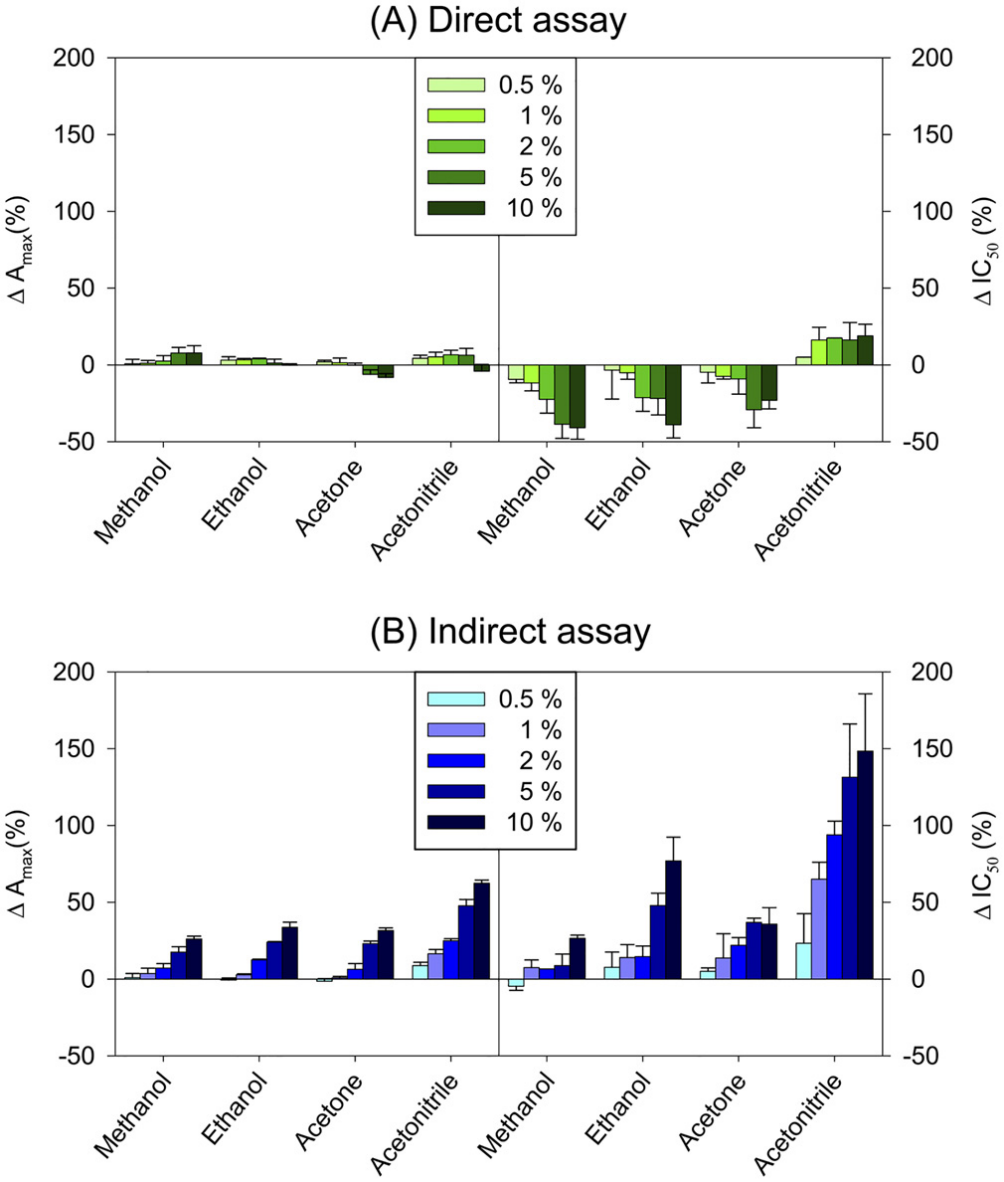
Variation (%) of A_max_ and IC_50_ values with the contents of organic solvents in the assay buffer. The white area sets the limits of acceptable working concentrations.

Once antibodies were evaluated in terms of affinity and specificity, competitive immunoassays were developed with the most sensitive antiserum (PQ*t*#2). The influence of different contents of common organic solvents (methanol, ethanol, acetone, and acetonitrile) over the inhibition curve parameters of the proposed immunoassays (see above) was studied (Fig 3).

Variations in A_max_ and IC_50_ values lower than 20% were deemed as acceptable. Accordingly, satisfactory A_max_ values in the direct cELISA were found for all evaluated solvents at concentrations up to 10%, whereas IC_50_ values significantly increased at concentrations higher than 2%, with acetonitrile arising as the most tolerated solvent. Concerning the indirect assay, methanol was the solvent with a lower influence over calibration curve parameters—concentrations lower than 10% had essentially no effect. Ethanol and acetone can be employed up to 5% and 2%, respectively, while acetonitrile strongly affects the indirect assay, so its use should be kept to a minimum or even avoided. All together, these results show that the direct assay is more robust and tolerates better the presence of organic solvents than the indirect assay. The direct cELISA seems to be particularly adequate for the analysis of solid samples, which usually require acetonitrile extraction prior to analysis, whereas the indirect assay might be more suitable for the analysis of alcohol-containing food matrices, such as beer, wine, or spirit samples.

**Fig 4:**
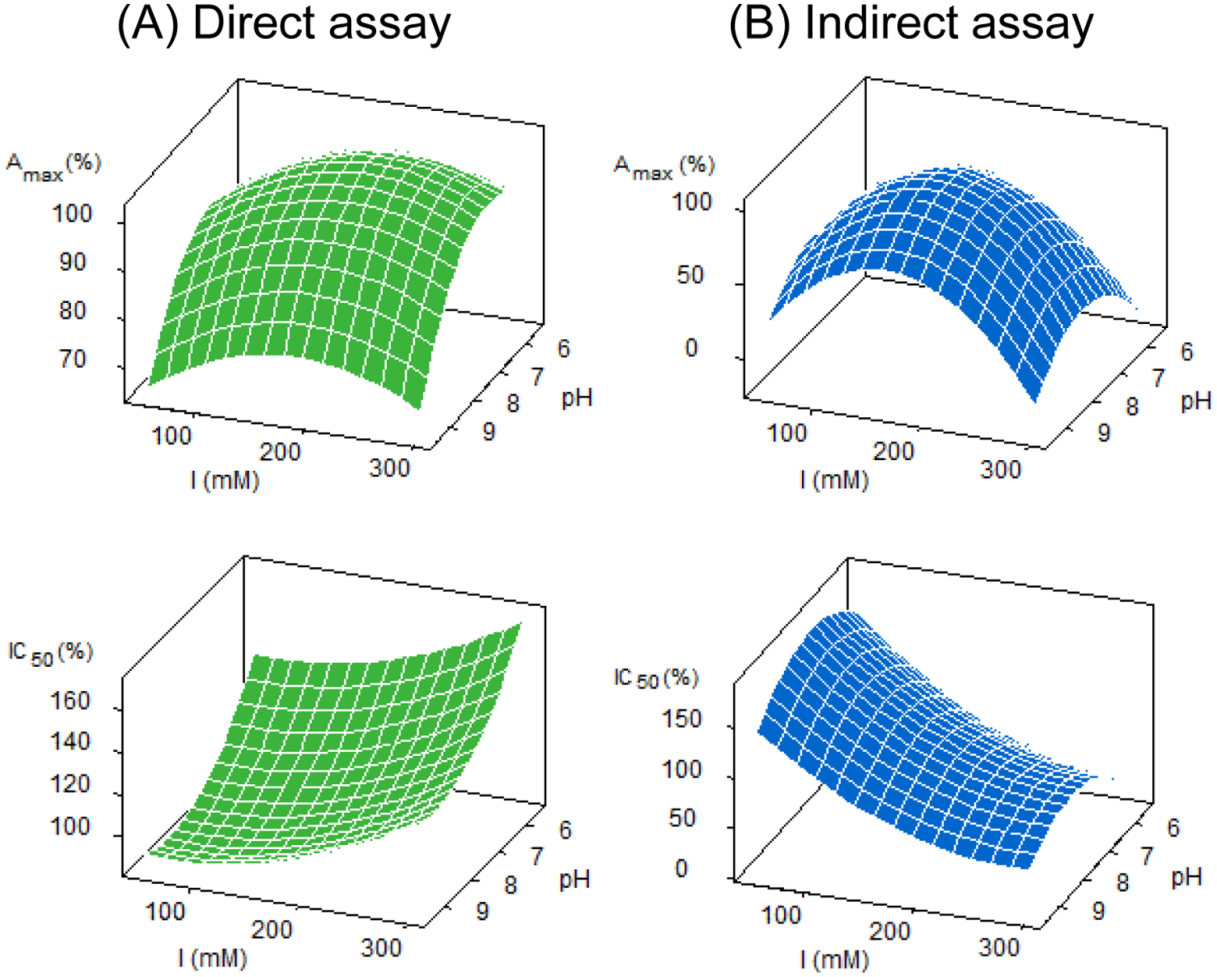
Variation (%) of A_max_ and IC_50_ values with buffer pH and ionic strength (*I*).

The inhibition curves obtained using buffers at different pH and ionic strength (*I*) values perfectly fitted to a sigmoidal equation. However, in the case of extreme pH and *I* conditions, analytical parameters deviated from those found at neutral conditions (Fig 4). Thus, in the case of the direct cELISA, A_max_ decreased at basic pH, while IC_50_ increased using buffers with low pH and high *I* values. Regarding the indirect format, A_max_ decreased under any condition slightly differing from PBS-T buffer, and IC_50_ was strongly influenced by the concentration of salts in the assay buffer. As it was also observed when studying the influence of organic solvents, it can be concluded that the proquinazid direct cELISA was more tolerant to changes in the buffer composition than the indirect assay.

### Analysis of samples

**Fig 5:**
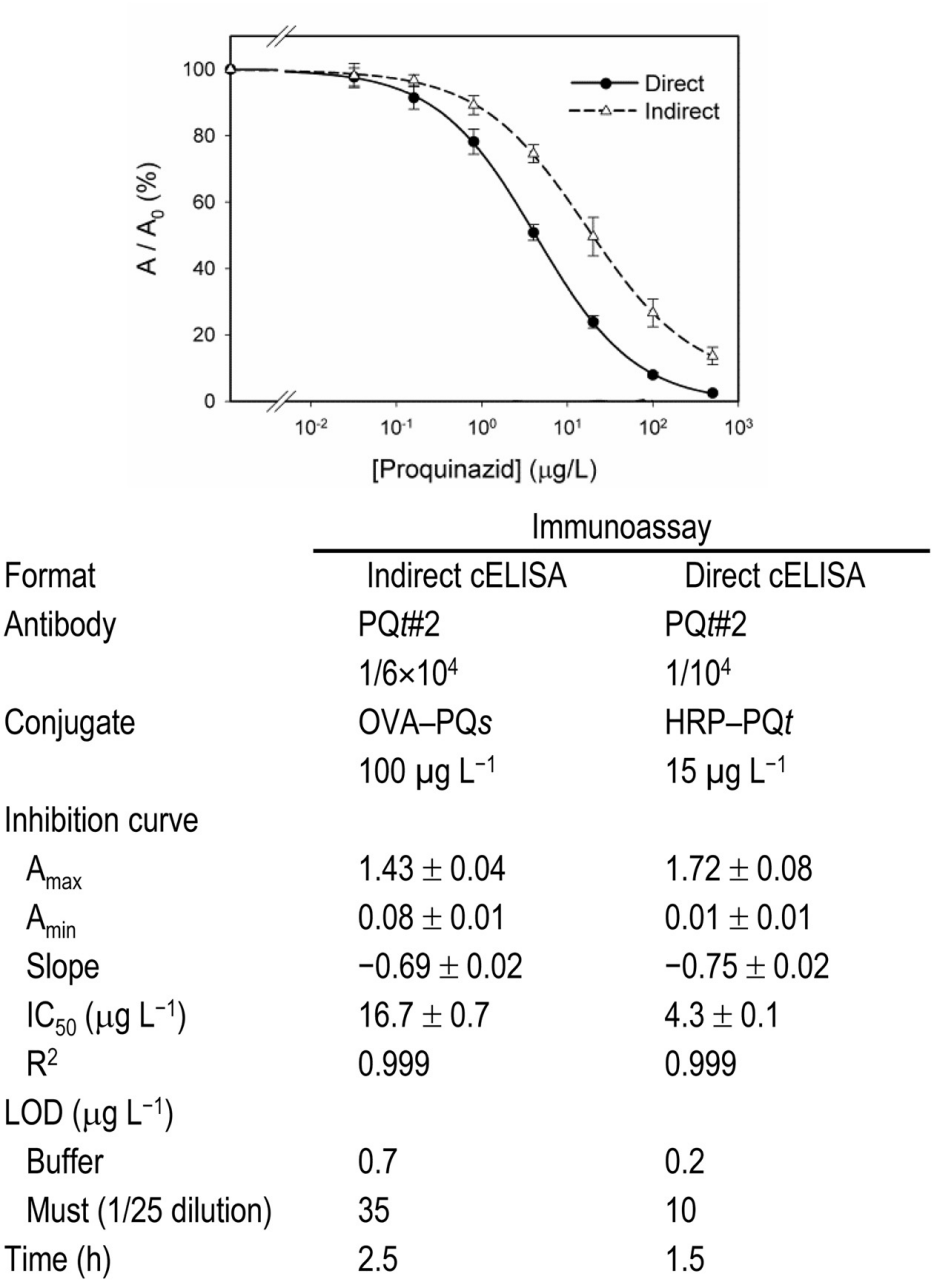
Conditions and parameters of the optimized immunoassays. Values are the mean of four independent experiments.

Final assay conditions, parameters of the inhibition curves, and other analytical properties of the proposed immunoassays are shown in Fig 5. The limit of detection (LOD) was estimated as the IC_10_ value of the inhibition curve (analyte concentration that provided a 10% inhibition of A_max_), with values of 0.2 and 0.7 μg/L for the direct and the indirect cELISA, respectively.

Since proquinazid is a highly recommended product against powdery mildew infestation in vineyards, white and red grape musts were selected as model commodities in order to evaluate the analytical performance of the proposed antibody-based assays. With this aim, commercial must samples were firstly diluted with deionized water (1/5, 1/25, 1/50, 1/250, and 1/500), and proquinazid standard curves were prepared in every diluted matrix and run in the optimized direct and indirect immunoassays. In both formats, inhibition curves made in must diluted 1/25 and those prepared in buffer were undistinguishable (results not shown), so this minimum dilution factor was chosen for further work in order to diminish matrix effects.

Recovery studies with the optimized cELISAs were performed using red and white must samples which had been spiked with proquinazid at 10, 50, 100, and 1000 μg/L. Adequate recovery values were found for grape musts down to 10 and 50 μg/L for direct and indirect cELISAs, respectively (Table 3). Concerning precision, relative standard deviations (RSDs) lower than 20% were obtained with both immunoassays.

**Table 3:** Recovery values and relative standard deviations (RSD) obtained in the analysis of spiked grape must samples by direct and indirect cELISAs with antibody PQ*t*#2.

Sample	Spiked PQ^a^ (μg/L)	Direct cELISA			Indirect cELISA		
		[PQ] (μg/L ± s, n = 3)	Recovery (%)	RSD (%)	[PQ] (μg/L ± s, n = 3)	Recovery (%)	RSD (%)
White must	10	8 ± 2	84	19.9	--^b^	--	--
	50	47 ± 3	94	7.1	49 ± 3	97	6.0
	100	94 ± 12	94	12.6	106 ± 10	106	10.7
	1000	940 ± 80	94	8.1	862 ± 90	86	10.1
Red must	10	10 ± 2	104	17.5	--	--	--
	50	49 ± 1	98	2.6	50 ± 8	101	16.8
	100	104 ± 4	104	3.6	88 ± 9	88	9.7
	1000	960 ± 60	96	6.4	820 ± 60	82	7.4

^a^ PQ stands for proquinazid.

^b^ Below assay detectability

Finally, blind-spiked grape musts were prepared and determined by the proposed direct and indirect cELISAs, as well as by a reference procedure based in QuEChERS extraction and GC‒MS analysis (Table 4). Comparison of the results obtained by each cELISA with those obtained by the instrumental method was performed using Deming method, which takes into account the experimental errors of every method (MedCalc Software bvba, Ostend, Belgium). The obtained regression curves were: [PQ]_Direct cELISA_ = (2 ± 5) + (1.00 ± 0.05) [PQ]_GC‒MS_ (n = 16), and [PQ]_Indirect cELISA_ = (−5 ± 10) + (0.98 ± 0.04) [PQ]_GC‒MS_ (n = 12). In both cases, results showed that there were not significant differences between the slope and 1 and between the intercept and 0, at a 95% confidence level, so we could conclude that cELISAs results were statistically comparable to those of the GC‒MS method.

**Table 4:** Analysis of proquinazid contents in blind-spiked must samples by chromatographic and immunochemical methods.

	[proquinazid] (μg L^−1^ ± s, n = 3)		
Sample^a^	GC—MS	Indirect cELISA	Direct cELISA
W1	12 ± 1	14 ± 3	---^b^
W2	18 ± 1	27 ± 9	---
W3	22 ± 2	28 ± 4	---
W4	53 ± 4	60 ± 10	70 ± 20
W5	80 ± 4	84 ± 8	100 ± 20
W6	200 ± 20	211 ± 8	200 ± 20
W7	420 ± 20	390 ± 70	450 ± 50
W8	530 ± 30	470 ± 70	500 ± 40
R1	47 ± 2	46 ± 7	---
R2	51 ± 3	55 ± 6	50 ± 10
R3	82 ± 5	83 ± 9	60 ± 10
R4	200 ± 10	200 ± 9	120 ± 30
R5	240 ± 20	223 ± 8	190 ± 20
R6	410 ± 30	430 ± 60	340 ± 60
R7	610 ± 30	620 ± 20	610 ± 40
R8	570 ± 40	620 ± 90	540 ± 30

^a^ The letter “W” refers to must samples from white grapes, whereas the letter “R” refers to must samples from red grapes.

^b^ Below LOD.

## Conclusions

Taking advantage of the Sonogashira cross-coupling reaction, two functionalized derivatives of proquinazid were synthesized by replacing the aryl iodine atom by an alkynyl carboxylate hydrocarbon chain and the corresponding saturated one—haptens PQ*t* and PQ*s*, respectively—in order to figure out whether these mimics were suitable haptens for specific and high-affinity antibody generation to this model compound. Following rabbit immunization, antibodies able to efficiently bind proquinazid were elicited from both haptens, thus demonstrating the suitability of this simple and efficient synthetic strategy for immunoreagent preparation to compounds bearing an aryl halide. Importantly, the haptens and antibodies herein described are the first ever reported immunoreagents for proquinazid analysis. Following antibody characterization and assay development, competitive immunoassays were employed to determine proquinazid residues in grape musts, and their analytical performance was satisfactorily validated by comparison with GC—MS.
